# Fucoxanthin, a Marine Carotenoid, Reverses Scopolamine-Induced Cognitive Impairments in Mice and Inhibits Acetylcholinesterase *in Vitro*

**DOI:** 10.3390/md14040067

**Published:** 2016-03-25

**Authors:** Jiajia Lin, Ling Huang, Jie Yu, Siying Xiang, Jialing Wang, Jinrong Zhang, Xiaojun Yan, Wei Cui, Shan He, Qinwen Wang

**Affiliations:** 1 Ningbo Key Laboratory of Behavioral Neuroscience, Zhejiang Provincial Key Laboratory of Pathophysiology, School of Medicine, Ningbo University, Ningbo 315211, China; linjiajiamed@yahoo.com (J.L.); nbhl1226@yahoo.com (L.H.); xiao.tai.yang_jie@163.com (J.Y.); xiangsi.ying@163.com (S.X.); wjl58452@yahoo.com (J.W.); 2 School of Marine Sciences, Ningbo University, Ningbo 315211, China; zhangjinrong@nbu.edu.cn (J.Z.); Yanxiaojun@nbu.edu.cn (X.Y.)

**Keywords:** fucoxanthin, scopolamine, acetylcholinesterase, Alzheimer’s disease, cognitive impairments

## Abstract

Fucoxanthin, a natural carotenoid abundant in edible brown seaweeds, has been shown to possess anti-cancer, anti-oxidant, anti-obesity and anti-diabetic effects. In this study, we report for the first time that fucoxanthin effectively protects against scopolamine-induced cognitive impairments in mice. In addition, fucoxanthin significantly reversed the scopolamine-induced increase of acetylcholinesterase (AChE) activity and decreased both choline acetyltransferase activity and brain-derived neurotrophic factor (BDNF) expression. Using an *in vitro* AChE activity assay, we discovered that fucoxanthin directly inhibits AChE with an IC_50_ value of 81.2 μM. Molecular docking analysis suggests that fucoxanthin likely interacts with the peripheral anionic site within AChE, which is in accordance with enzymatic activity results showing that fucoxanthin inhibits AChE in a non-competitive manner. Based on our current findings, we anticipate that fucoxanthin might exhibit great therapeutic efficacy for the treatment of Alzheimer’s disease by acting on multiple targets, including inhibiting AChE and increasing BDNF expression.

## 1. Introduction

Alzheimer’s disease (AD) is one of the most common neurodegenerative disorders leading to cognitive impairments [1]. Although the etiology of this disease is not precisely known, many factors, including cholinergic system dysfunction, β-amyloid (Aβ) deposits and oxidative stress, have been considered to play important roles in the occurrence and development of AD [2]. Currently, the primary treatment for AD, which only provides symptomatic relief, is a cholinergic replacement therapy, represented by four FDA-approved acetylcholinesterase (AChE) inhibitors, donepezil, galantamine and rivastigmine [3].

Scopolamine is an antagonist of the muscarinic acetylcholine receptor. Many studies have shown that a high dose of scopolamine impairs short-term learning and memory in animal models and humans [4,5,6]. Therefore, scopolamine has become widely accepted as an experimental model of AD and is used to screen for anti-amnesic drugs [7,8]. Scopolamine induces cognitive impairment associated with an attenuation of cholinergic neurotransmission, as well as increased oxidative stress and inflammation in the brain [9,10]. Therefore, drugs that can effectively enhance cholinergic transmission and/or reduce oxidative stress and inflammation might reverse scopolamine-induced cognitive impairments [11].

Marine carotenoids are widely present in both plants and animals. Many marine carotenoids, including fucoxanthin, astaxanthin and lutein, are reported to produce anti-oxidant, anti-inflammatory, anti-obesity and anti-diabetic effects [12,13]. Fucoxanthin is the most abundant marine carotenoid and contributes more than 10% of the total production of carotenoids in nature [13]. Fucoxanthin contains unique functional groups, including an unusual allenic bond and a 5,6-monoepoxide structure [14]. Previous studies have shown that fucoxanthin exhibits various health benefits [15,16,17]. For example, fucoxanthin in diet significantly reduced weight gain in experimental animals by increasing fatty acid oxidation and heart production in white adipose tissue [18]. Moreover, rats that were fed with fucoxanthin displayed decreased levels of oxidative stress markers and increased activities of antioxidant enzymes [19].

Interestingly, a recent study has also shown that fucoxanthin can ameliorate Aβ-induced oxidative stress in microglia cells, suggesting that fucoxanthin might be useful for the treatment of AD [20]. In this study, we first evaluated the effects of fucoxanthin on scopolamine-induced cognitive impairments in mice. We further examined if fucoxanthin could directly inhibit AChE *in vitro*. Finally, we investigated the molecular basis of the interaction between fucoxanthin and AChE by molecular docking simulation.

## 2. Results

### 2.1. Fucoxanthin Does Not Affect Locomotor Activity in the 5-min Open-Field Test

**Figure 1:**
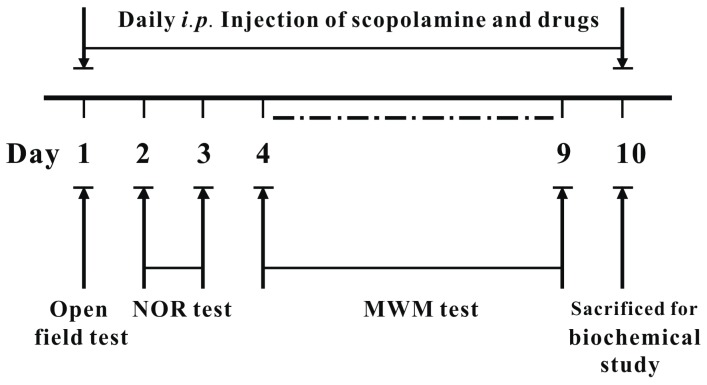
Experimental design and schedule of animal tests. NOR, novel object recognition; MWM: Morris water maze.

**Figure 2:**
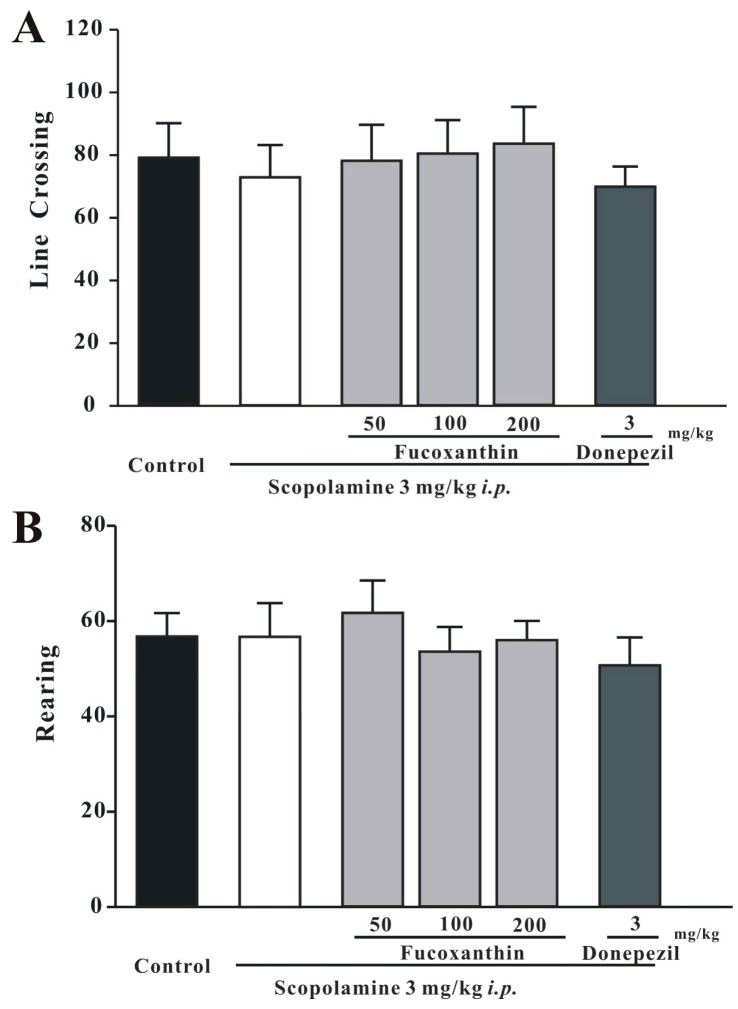
Treatments do not affect locomotor activity in the 5-min open field tests. The number of line crossings and rearings in the open field test are shown in (**A**) and (**B**), respectively. Data are expressed as the mean ± SEM (*n* = 8).

To examine whether fucoxanthin could reverse cognitive impairments in an animal model, we used the scopolamine-induced cognitive impairment mouse model and evaluated its effects with the open-field, novel object recognition (NOR) and Morris water maze tests. The schedule of animal experiments is shown in Figure 1. Drugs were given 30 min prior to the test each day. Locomotor activity was examined by testing the number of line crossings and rearings in the open-field test for 5 min. As shown in Figure 2, none of the treatments significantly altered the number of line crossings or rearings (for line crossing, one-way ANOVA, *F*(5, 42) = 0.784, *p* > 0.05; for rearing, *F*(5, 42) = 1.443, *p* > 0.05).

### 2.2. Fucoxanthin Reverses Scopolamine-Induced Recognition Impairment in the NOR Test

**Figure 3:**
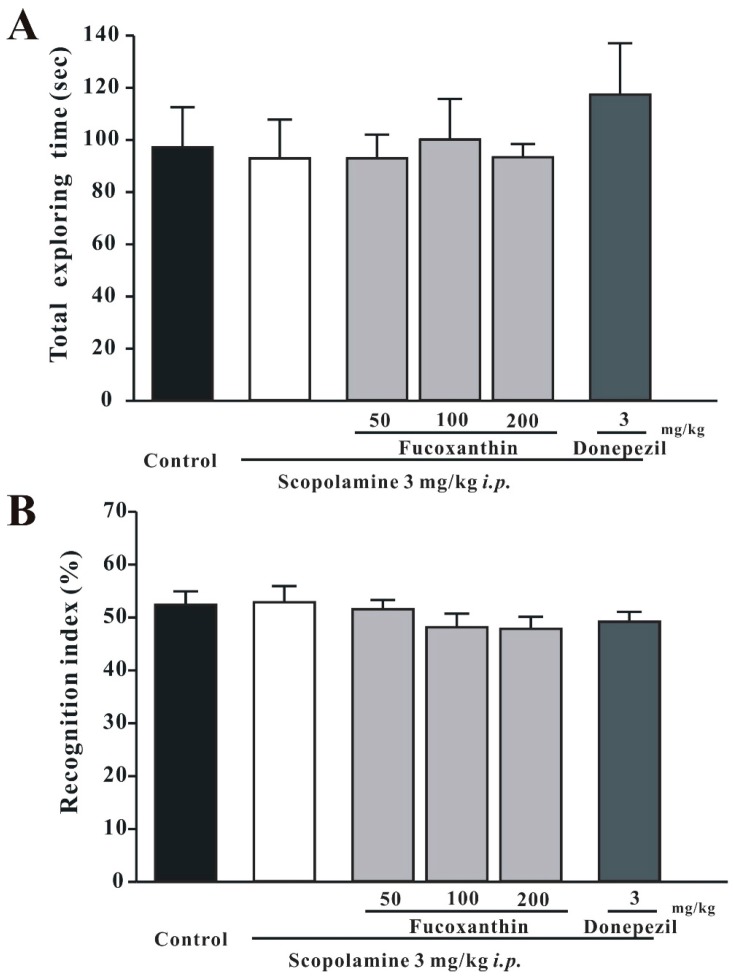
In the training session, all groups were found to possess similar total exploring times and recognition indexes for two identical objects. The total exploring times and recognition indexes in the training session are shown in (**A**) and (**B**), respectively. Data are expressed as the mean ± SEM (*n* = 8).

The NOR test was used to evaluate whether fucoxanthin could reverse scopolamine-induced recognition impairment. On the first day of the NOR test, mice were habituated to the experimental arena without any behaviorally-relevant stimulus. The training session was conducted one day after the habituation session. The exploration time of two identical objects was recorded in the training sessions 30 min after drug treatments. In this session, all groups exhibited similar total exploring times and recognition indexes for both objects (for total exploring times, one-way ANOVA, *F*(5, 42) = 0.672, *p* > 0.05; for the recognition index, *F*(5, 42) = 1.513, *p* > 0.05; Figure 3).

**Figure 4:**
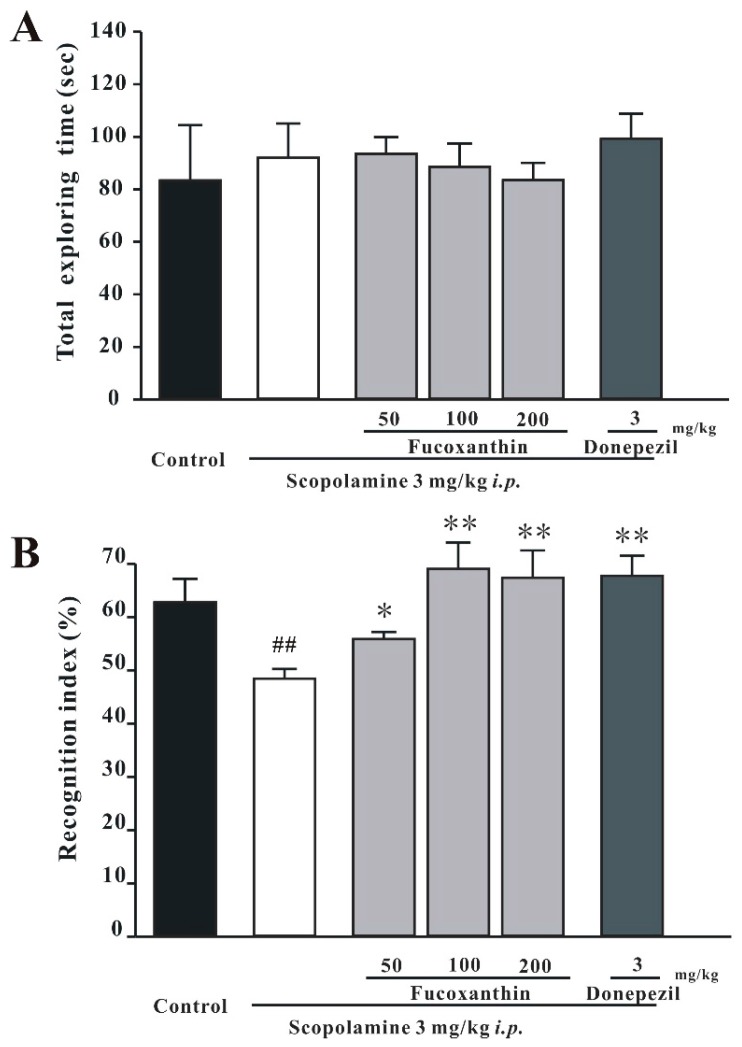
Fucoxanthin reverses scopolamine-induced recognition impairments in mice. The total exploring time and recognition index in the retention session are shown in (**A**) and (**B**), respectively. Data are expressed as the mean ± SEM (*n* = 8; ^##^ *p* < 0.01 *versus* the control group, * *p* < 0.05 and ** *p* < 0.01 *versus* the scopolamine-treated group (one-way ANOVA and Tukey’s test)).

The retention session was conducted one day after the training session. Although the total exploration time was similar among groups (one-way ANOVA, *F*(5, 42) = 0.434, *p* > 0.05; Figure 4A), the recognition index for the novel object was significantly changed (one-way ANOVA, *F*(5, 42) = 12.966, *p* < 0.01; Figure 4B). The recognition index in the control mice was significantly higher than that in the scopolamine-induced mice (Tukey’s test, *p* < 0.01). Moreover, treatment with fucoxanthin or donepezil significantly reversed the scopolamine-induced decrease of the recognition index (Tukey’s test, *p* < 0.01).

### 2.3. Fucoxanthin Reverses Scopolamine-Induced Spatial Learning and Memory Impairments in the Morris Water Maze Test

**Figure 5:**
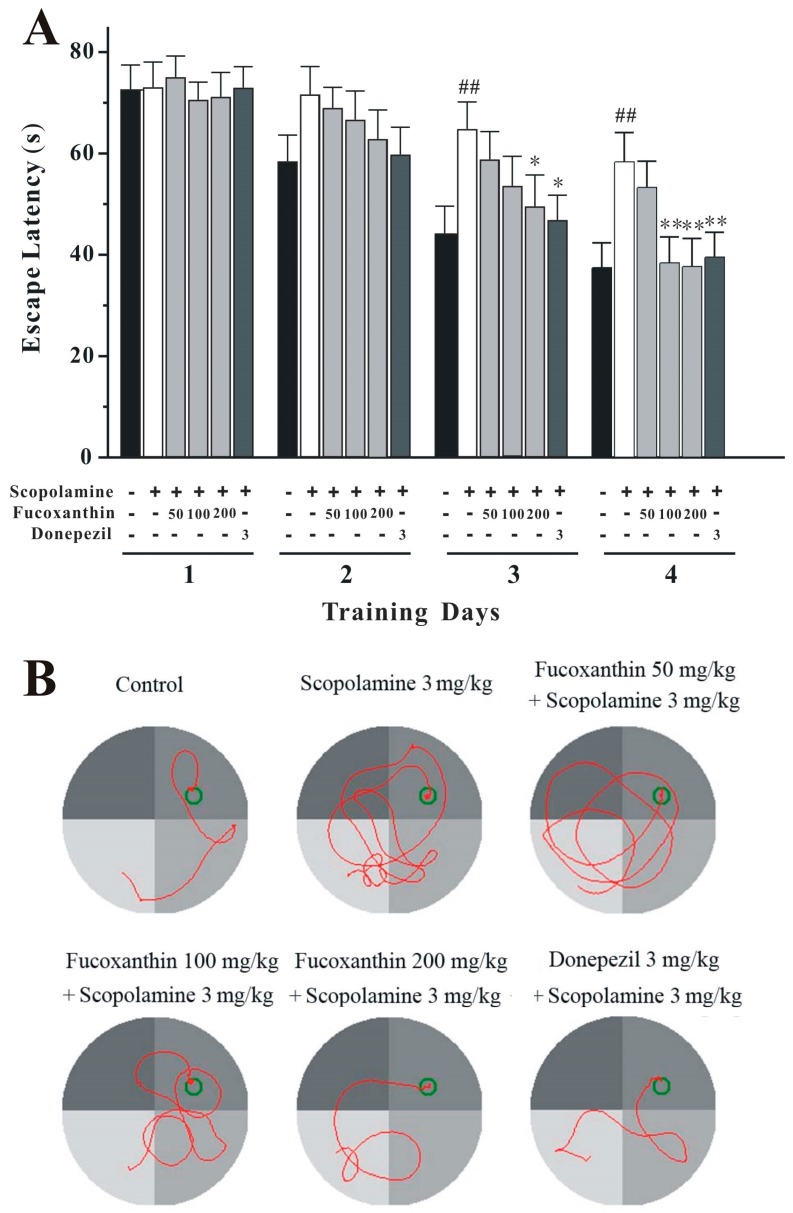
Fucoxanthin reverses scopolamine-induced spatial learning and memory deficits in the training session of the Morris water maze task. (**A**) Mean latencies to escape from the water onto the hidden platform in training trials. Each mouse was subjected to four trials per day for four consecutive days. Data are expressed as the mean ± SEM (*n* = 8); ^##^ *p* < 0.01 *versus* the control group, * *p* < 0.05 and ** *p* < 0.01 *versus* the scopolamine-treated group (two-way repeated-measures ANOVA and LSD test). (**B**) Representative swimming-tracking paths of various groups as indicated in the training session.

We further studied whether fucoxanthin could reverse scopolamine-induced spatial learning and memory impairments using the Morris water maze. Two-way repeated-measures ANOVA revealed significant changes in treatment effect (two-way ANOVA *F*(5, 168) = 6.333, *p* < 0.01) and time effect (*F*(3, 168) = 9.211, *p* < 0.01), but not for the treatment × time interaction (*F*(15, 168) = 0.717, *p* > 0.05; Figure 5A). The performance of mice in all groups improved throughout the training session, as indicated by the shortened escape latency during this period (Figure 5A). The scopolamine-treated group took a significantly longer time to find the platform on the last two days of training when compared to the control group, suggesting that scopolamine causes spatial learning impairments (*p* < 0.01; Figure 5). Fucoxanthin at medium and high doses significantly decreased the scopolamine-induced increase of the mean latency on the third and fourth day of the training session (*p* < 0.05; Figure 5). Under the same conditions, donepezil also reserved the scopolamine-elevated mean latency on the last two days of training (*p* < 0.05; Figure 5).

**Figure 6:**
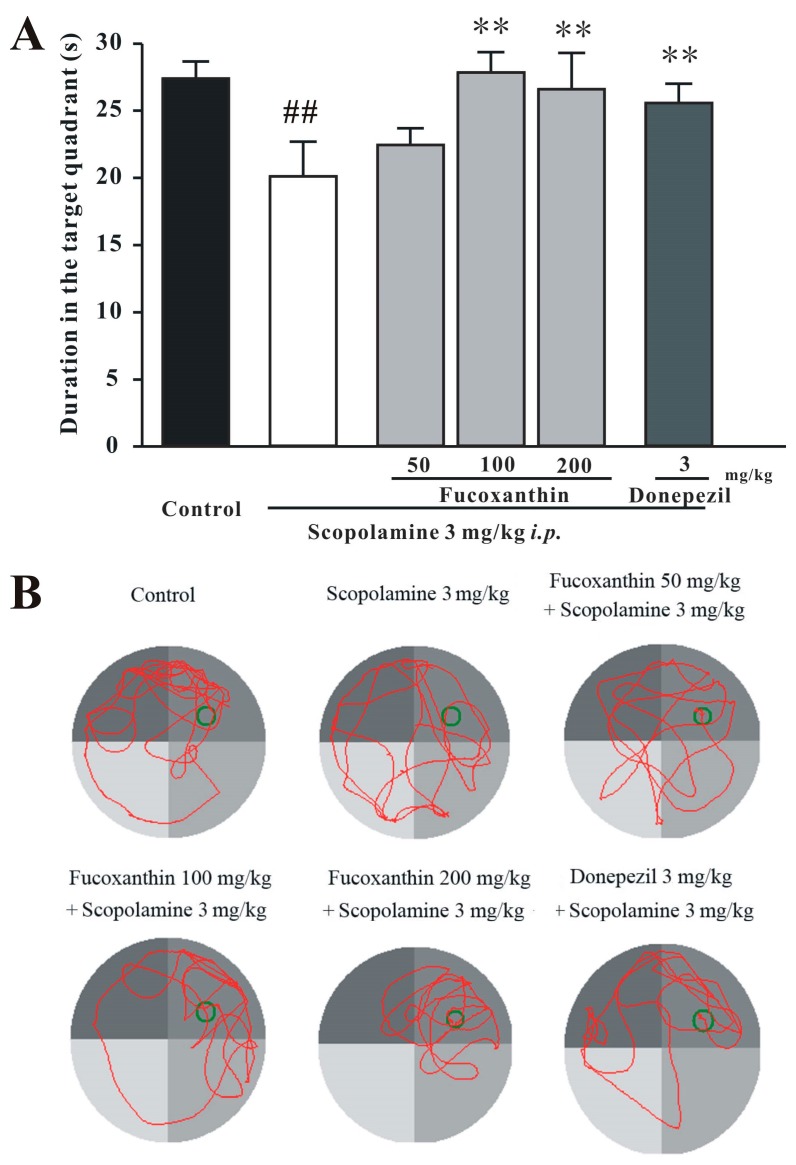
Fucoxanthin reverses scopolamine-induced spatial learning and memory deficits in the probe trial of the Morris water maze task. (**A**) The swimming time in the target quadrant (in which the platform had been placed during the training session) in the probe trial (swimming 90 s without platform). Data are expressed as the mean ± SEM (*n* = 8); ^##^ *p* < 0.01 *versus* the control group, ** *p* < 0.01 *versus* the scopolamine-treated group (ANOVA and Tukey’s test). (**B**) Representative swimming-tracking paths of various groups as indicated in the probe trials are demonstrated.

In the probe trial, the time spent in the target quadrant was significantly different among groups (one-way ANOVA, *F*(5, 42) = 13.415, *p* < 0.01; Figure 6). Scopolamine significantly decreased the time spent in the target quadrant when compared to the control group (Tukey’s test, *p* < 0.01; Figure 6). However, fucoxanthin (100 and 200 mg/kg) or donepezil caused a significant increase in the time spent in the target quadrant when compared to scopolamine alone (Tukey’s test, *p* < 0.01; Figure 6).

### 2.4. Fucoxanthin Increases the Expression of BDNF in the Hippocampus and Cortex of Mice

**Figure 7:**
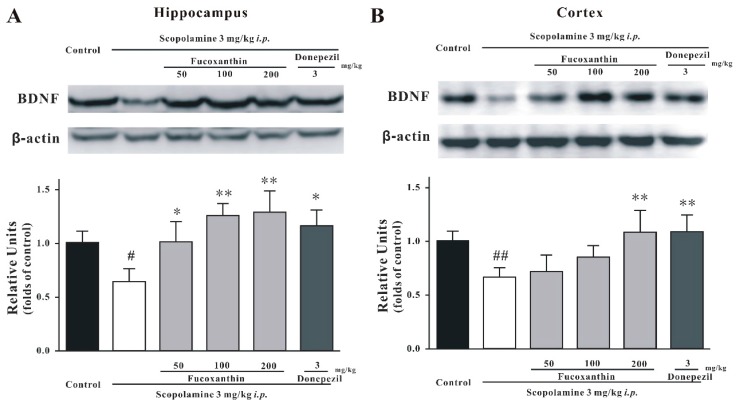
Fucoxanthin significantly reverses the scopolamine-induced decrease in the expression of BDNF in mice. BDNF expression in (**A**) the hippocampus and (**B**) the cortex of mice was detected by Western blotting. Data are expressed as the mean ± SEM (*n* = 3); ^#^ *p* < 0.05 and ^##^ *p* < 0.01 *versus* the control group, * *p* < 0.05 and ** *p* < 0.01 *versus* the scopolamine-treated group (ANOVA and Tukey’s test).

Brain-derived neurotrophic factor (BDNF) levels in the brains of mice were detected by Western blot analysis. On the 10th day of treatment, the mice were sacrificed, and the hippocampus and cortex were dissected. BDNF levels of the control group were significantly higher than those of the scopolamine-treated group in both the hippocampus and cortex (Tukey’s test, *p* < 0.05; Figure 7). Moreover, fucoxanthin significantly increased the expression of BDNF in the hippocampus and cortex (Tukey’s test, *p* < 0.05; Figure 7). Under the same conditions, donepezil also reversed the scopolamine-induced decrease of BDNF expression (Tukey’s test, *p* < 0.05; Figure 7).

### 2.5. Fucoxanthin Increases ChAT Activity and Decreases AChE Activity in the Hippocampus and Cortex of Mice

Choline acetyltransferase (ChAT) and AChE activities were also measured on the 10th day of treatment. As shown in Table 1, compared to the control group, ChAT and AChE activities in both the hippocampus and the cortex were significantly changed in the scopolamine-induced groups (Tukey’s test, *p* < 0.05; Table 1 and Table 2). Notably, both fucoxanthin (100 and 200 mg/kg) and donepezil significantly reduced the scopolamine-induced increase of AChE activity in the hippocampus and cortex (Tukey’s test, *p* < 0.01; Table 1). Moreover, fucoxanthin (100 and 200 mg/kg) and donepezil also significantly reversed the scopolamine-induced decrease of ChAT activity in the hippocampus and cortex (Tukey’s test, *p* < 0.05; Table 2).

### 2.6. Fucoxanthin Directly Inhibits AChE in a Non-Competitive Manner in Vitro

**Figure 8:**
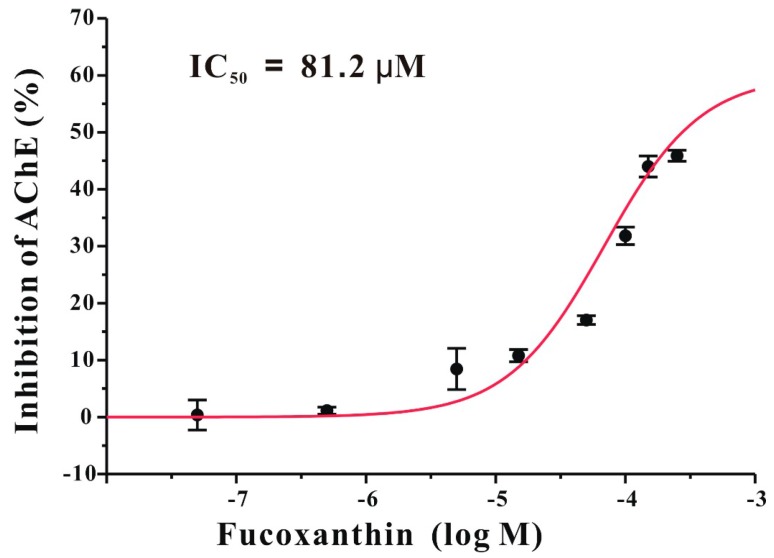
Fucoxanthin directly inhibits AChE enzyme activity in a concentration-dependent manner. The inhibitory effect of fucoxanthin on AChE is shown in the graph. The IC_50_ value is also indicated in the graph. Data are expressed as the mean ± SEM (*n* = 3).

**Figure 9:**
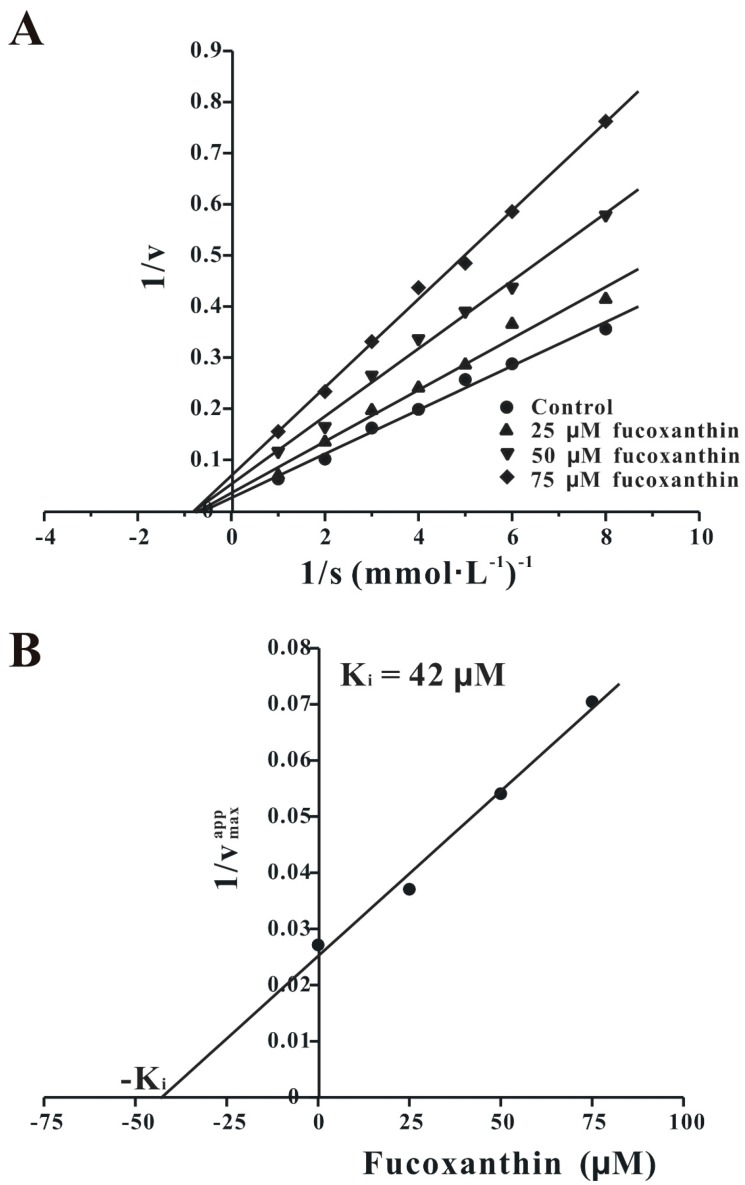
Fucoxanthin inhibits AChE by a non-competitive mechanism. (**A**) Kinetic analysis of AChE inhibition by fucoxanthin. The AChE enzyme was assayed in either the presence (25, 50 or 75 μM) or absence of fucoxanthin over a range of concentrations of acetylthiocholine iodide (0.125–1 mM). The ranges of 1/v *versus* 1/[S] were fitted by a Lineweaver-Burk plot. The data are expressed as the mean of three independent experiments. (**B**) The K_i_ value of fucoxanthin in the inhibition of AChE.

Our *in vivo* results have shown that fucoxanthin could effectively reverse scopolamine-induced cognitive impairments. Because AChE inhibitors used for AD treatment were reported to have similar effects, we speculated that fucoxanthin may also directly inhibit AChE [11]. To test this, we used an *in vitro* AChE activity assay. In our study, fucoxanthin directly inhibits AChE with an IC_50_ of 81.2 μM (Figure 8). To further explore the mode of AChE inhibition by fucoxanthin, three concentrations (25, 50 or 75 μM) of fucoxanthin were used in the *in vitro* AChE activity assay containing various concentrations (0.125–1 mM) of substrate. Analysis of these assays with Lineweaver–Burk plots shows that fucoxanthin acts as a non-competitive inhibitor of AChE, and the K_*i*_ value of AChE inhibition by fucoxanthin is 42 μM (Figure 9).

### 2.7. Molecular Docking Analysis of the Interaction between Fucoxanthin and AChE

**Figure 10:**
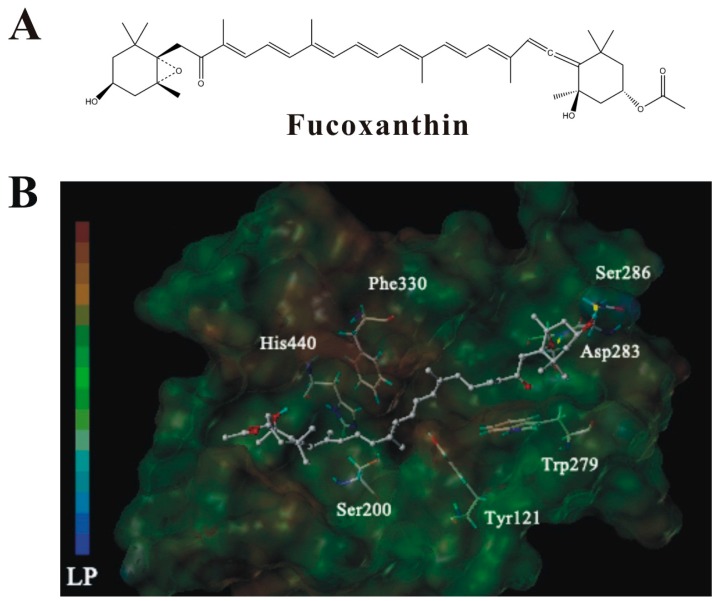
Molecular docking simulation of the interaction between AChE and fucoxanthin. (**A**) Chemical structure of fucoxanthin; (**B**) low-energy conformation of fucoxanthin mainly bound to the peripheral anionic site (PAS) of AChE generated by molecular docking. Fucoxanthin is depicted as a stick model showing carbon (white), oxygen (red) and hydrogen (green). Yellow line: hydrogen bond. LP: lipophilic potential.

To gain insight into the molecular interaction between fucoxanthin and AChE, computational docking was performed. Fucoxanthin showed favorable interaction mainly with the peripheral anionic site (PAS) of AChE (Figure 10). The Surflex-Dock score is expressed as −log (K_d_) (the unit of K_d_ is M). The Surflex-Dock score, evaluated automatically by the software, was 4.74. Therefore, the value of K_d_ could be easily calculated and equals 1.8 × 10^−5^ M (18 μM), which is close to the experimental value determined by our *in vitro* activity assay. Moreover, the docking analysis suggests that fucoxanthin might form hydrogen bonds with Asp283 and Ser286, respectively, in the PAS of AChE.

## 3. Discussion

In the current study, we have reported for the first time that fucoxanthin, a carotenoid extracted from brown seaweeds, can effectively reverse scopolamine-induced impairments of cognition in mice, suggesting that fucoxanthin might be used in the treatment of AD. We have further demonstrated that fucoxanthin significantly increases ChAT activity and BDNF expression and decreases AChE activity in the brains of scopolamine-treated mice. Moreover, fucoxanthin directly inhibits AChE in a non-competitive manner *in vitro*, possibly via interacting with the PAS of AChE.

Fucoxanthin is a compound safe for use in humans and animals [21]. Previous studies have shown that repeated oral administration of fucoxanthin at 1000 mg/kg for 30 days in ICR mice resulted in no mortality, no abnormalities in gross appearance and no abnormal findings in histological observations [21,22]. Moreover, oral administration of fucoxanthin at 50–200 mg/kg exhibited anti-cancer, anti-obesity and anti-diabetic affects *in vivo* [15,16,17]. Therefore, we tested the effects of repeated oral administration of fucoxanthin at 50–200 mg/kg on cognitive impairments in our study.

Administration of scopolamine in animals produces short-term learning and memory deficits, which are considered to be characteristic of cholinergic dysfunction in AD [4,5,6]. Therefore, the scopolamine-induced amnesic model has been widely accepted as a pharmacological model of cognitive impairments useful for screening potential cognitive-enhancing agents. In our study, fucoxanthin effectively reversed scopolamine-induced recognition impairments in the NOR test and spatial learning and memory impairments in the Morris water maze test, suggesting that fucoxanthin has cognitive-enhancing potential. BDNF is a neurotrophin that facilitates synaptic transmission and neuronal plasticity [23]. Importantly, BDNF plays an essential role in the formation and storage of memory. The expression of BDNF is downregulated not only in the brains of AD patients, but also in amnesic animal models, including the scopolamine-induced cognitive impairment model [11]. In our study, fucoxanthin significantly reversed the reduction in BDNF expression caused by scopolamine treatment, suggesting that fucoxanthin might increase memory formation in animals by increasing BDNF levels.

Alternatively, decreased acetylcholine expression also generally results in a diminished ability to learn and form new memories. The concentration of acetylcholine in the brain is dynamically regulated by the activities of AChE and ChAT [24]. Therefore, we evaluated the activity of these two enzymes in the brains of our treated mice. In our study, scopolamine decreased ChAT activity and increased AChE activity in both the hippocampus and the cortex, which is in accordance with previous studies [9,10]. Interestingly, fucoxanthin significantly reversed the scopolamine-induced alterations of ChAT and AChE activities, suggesting that fucoxanthin might directly affect enzymes in the cholinergic system.

We further explored if fucoxanthin could directly inhibit AChE, a key enzyme in the cholinergic system. AChE hydrolyzes acetylcholine into acetic acid and choline, and AChE inhibitors prolong the action of acetylcholine synapses and enhance cholinergic neurotransmission [25]. Therefore, many AChE inhibitors are clinically used in the treatment of neurological diseases associated with cholinergic deficits, including AD in particular [26]. Our results show that fucoxanthin directly inhibits the activity of AChE *in vitro*. Moreover, Lineweaver–Burk plots suggest that fucoxanthin acts as a non-competitive inhibitor of AChE.

AChE possesses two important binding sites, the catalytic anion site (CAS) and the PAS [27]. The CAS and the PAS are responsible for catalytic activity and ligand binding, respectively. The PAS also serves as a binding site for non-competitive inhibitors [28]. By using molecular docking analysis, we investigated the interaction between fucoxanthin and AChE. In the simulated binding of fucoxanthin and AChE, the 5,6-monoepoxide structure of fucoxanthin formed two hydrogen bonds with Asp283 and Ser286 in the PAS of AChE. Therefore, our molecular docking analysis further supports our finding that fucoxanthin acts as a non-competitive inhibitor of AChE.

Why was the concentration of fucoxanthin (50–200 mg/kg) used in our study much higher than that of donepezil (3 mg/kg)? Donepezil is much more potent than fucoxanthin in inhibiting AChE *in vitro*. Therefore, it is rational to expect that a higher concentration of fucoxanthin than that of donepezil is needed to reverse AChE-involved scopolamine-induced cognitive impairments *in vivo*. Does fucoxanthin reverse scopolamine-induced cognitive impairments solely via the direct inhibition of AChE? Donepezil was reported to inhibit AChE with an IC_50_ around 10 nM [29]. However, in our study, fucoxanthin inhibited AChE with an IC_50_ of 81.2 μM. Interestingly, fucoxanthin at 100 mg/kg produced anti-cognitive impairment effects as strong as that produced by 3 mg/kg donepezil in our animal model. These results suggest that the beneficial effects of fucoxanthin in the scopolamine-induced cognitive impairments mouse model may not solely result from direct inhibition of AChE. We speculated that besides inhibition of AChE, fucoxanthin might act on additional targets that are involved in cognitive enhancement. A recent study has shown that fucoxanthin attenuates pro-inflammatory cytokine secretion, reduces reactive oxygen species formation and elevates anti-oxidative enzymes in Aβ-treated microglia cells [20]. Because oxidative stress and inflammation play important roles in scopolamine-induced cognitive impairments, it is rational to speculate that fucoxanthin might protect against cognitive impairments by reducing the scopolamine-induced increases in oxidative stress and inflammation. To further determine whether fucoxanthin protects against cognitive impairments via anti-oxidant and anti-inflammatory mechanisms, additional experiments, including analysis of pro-inflammatory cytokine production and anti-oxidative enzyme expression, are being undertaken in our lab. Moreover, it is agreed that several risk factors, including diabetes and obesity in particular, are related to AD [30]. Therefore, fucoxanthin with anti-diabetic and anti-obesity potential might also be helpful for the prevention of AD.

## 4. Materials and Methods

### 4.1. Chemicals and Reagents

Fucoxanthin was obtained from *Sargassum horneri*, a genus of brown seaweeds, by a purification method based on solvent extraction, low temperature concentration and ethanol precipitation. Briefly, fucoxanthin extraction was performed at 30 °C for 2 h with an ethanol to sample ratio of 4:1 mL/g. The extraction solution was then concentrated at 25 °C. The precipitation of lipid and chlorophylls further occurred when the concentration solution contained 63% ethanol. Fucoxanthin was then purified by precipitation when the solution reached an ethanol content of 40%. The purity of fucoxanthin was more than 80% by HPLC. Fucoxanthin was stored at −20 °C before use. Donepezil, scopolamine, dithiobisnitrobenzoic acid (DTNB), ethopropazine hydrochloride and acetylthiocholine iodide (ATCI) were purchased from Sigma-Aldrich (St. Louis, MO, USA).

### 4.2. Drug Treatment for Animal Study

Male Institute of Cancer Research (ICR) mice weighing 25–30 g were obtained from Zhejiang Academy of Medical Sciences. The ICR mouse is a strain of albino mice originating in Switzerland [31]. The animals were maintained on a 12-h light/dark cycle under controlled temperature (22 ± 2 °C) and humidity (50% ± 10%) and given standard diet and water. Animals were allowed to acclimatize for 3 days before the experiments. All procedures followed the National Institutes of Health (NIH) Guide for the Care and Use of Laboratory Animals (NIH Publications No. 80-23, revised 1996) and were approved by the Animal Care and Use Committee of Ningbo University (SYXK-2008-0110).

Fucoxanthin was dissolved in sterile saline containing 0.5% Tween-20. Donepezil and scopolamine were dissolved in sterile saline. Mice were randomly assigned into six groups of 8 animals each: control, 3 mg/kg scopolamine, 3 mg/kg scopolamine plus low (50 mg/kg), medium (100 mg/kg) and high (200 mg/kg) doses of fucoxanthin and 3 mg/kg scopolamine plus donepezil (3 mg/kg). Fucoxanthin was given by intragastric (*i.g.*) administration. Donepezil or scopolamine was given by intraperitoneal (*i.p.*) injection. All drugs were given 30 min prior to each trial once a day for 10 consecutive days. Mice were sacrificed for biochemical study on the 10th day. All animals received the last injection of drugs 30 min prior to being sacrificed.

### 4.3. Open-Field Test

To analyze the exploratory and locomotor activities, animals were placed in the left rear quadrant of a 50 × 50 × 39-cm open field with white plywood walls and a brown floor divided into 4 identical squares of equal dimensions (25 × 25 cm) [32]. The animals were placed one by one at the center of the box and allowed to explore it for 5 min. Hand-operated counters and stopwatches were used to score the number of line crossing with four paws and the number of rearings (number of times the animals stood on its hind legs), which were used as indicators of locomotor and exploratory activities, respectively. The person counting was blind to the drug status of the subjects. To avoid perturbation of the animals due to urine and feces, between two tests, the open field was cleaned with 70% ethanol solution and a dry cloth.

### 4.4. Novel Object Recognition Test

The NOR test was carried out in an open-field arena (30 × 30 × 30 cm) built with polyvinyl chloride plastic, plywood and transparent acrylic as described before [33,34]. The task consisted of three sessions: habituation, training and retention sessions that were carried out over a period of three consecutive days. On the first day, the animals were habituated to the experimental arena by allowing them to freely explore the arena for 5 min in the absence of any behaviorally-relevant stimulus. On the second day, the animals were allowed to explore two identical objects for 5 min. On the third day, one of the objects was changed to a novel one with a different shape and color, and the animals were allowed to explore the arena for 5 min. To avoid perturbation of the animals due to urine and feces, between two tests, the field was cleaned thoroughly with 70% ethanol solution and a dry cloth. Exploration was defined as sniffing or touching the objects with the nose and/or forepaws at a distance of less than 2 cm. Sitting on or turning around the objects closely was not considered exploratory behavior. The exploration was scored manually using a video camera positioned over the arena by an observer blind to testing conditions. Total exploring time is the amount of time spent exploring both objects. A recognition index, which is the ratio of the amount of time spent exploring either of the two objects (training session) or the novel object (retention session) over the total exploring time, was used to measure cognitive function.

### 4.5. Morris Water Maze Task

The Morris water maze task was carried out as described before [35]. The water maze apparatus consisted of a circular pool 110 cm in diameter, filled with water at 23 ± 2 °C to cover a platform. The platform always resided in the center of the northeast quadrant, except on the last day. Each mouse’s swimming was monitored by a video camera linked to a computer-based image analyzer. Learning performance was tested for 4 consecutive days beginning on the 5th day after the first injection of scopolamine. Each mouse was trained to find the platform with four trials per day. In each trial, the time required to escape onto the hidden platform was recorded. On the 9th day, a probe trial was made by removing the platform and allowing the mice to swim for 90 s in search of it. Swimming time in each of the four quadrants in the pool was calculated. A persistent preference for the quadrant previously occupied by the platform was taken to indicate that the mice had acquired and remembered the spatial task.

### 4.6. Western Blot Analysis

Western blot analysis was performed as previously described [36]. Briefly, after decapitation, the brains of mice were removed quickly. Half of the brain tissue was analyzed by Western blot analysis, and the other half was analyzed by AChE and ChAT activity assays. For Western blot analysis, the hippocampi and cortex were dissected on ice. Brain tissue samples were homogenized at 4 °C for 1 min in lysis buffer (50 mM Tris-HCl, 150 mM NaCl, 1% Triton X-100, 1 mM EDTA, 1 mM phenylmethanesulfonyl fluoride, 0.1% sodium dodecyl sulfate, 1% sodium deoxycholate, 5 µg/mL leupeptin, 1 µg/mL aprotinin and 5 µg/mL pepstatin). After centrifugation at 16,000× *g* for 10 min, the protein concentration in the supernatant was determined by the Bradford assay. Samples (40 µg) were separated by SDS-PAGE and transferred to polyvinylidene fluoride membranes for 2 h at 100 V. The membranes were further blocked with 5% non-fat milk in PBST (0.1% Tween 20 in phosphate-buffered saline) for 2 h. The blots were incubated overnight at 4 °C with antibodies against BDNF (1:500, Santa Cruz Biotechnology, Santa Cruz, CA, USA) and β-actin (1:1000, Santa Cruz Biotechnology). After three washes with PBST, the membranes were further incubated with secondary antibody. Blots were developed by using an enhanced chemiluminescence plus kit (Amersham Bioscience, Aylesbury, UK) and then exposed to Kodak films. All data are the results from three independent experiments. Data were expressed as a ratio to the optical density (OD) values of control samples for statistical analyses.

### 4.7. Measurement of Choline ChAT Activity ex Vivo

Mouse brain was weighted, followed by the addition of a 10-times volume of lysis buffer (10 mM HEPES, pH 7.5, 1 mM EDTA, 1 mM EGTA, 150 mM NaCl and 0.5% Triton X-100). Homogenization was then performed by vortexing on ice for 15 min. The homogenates were clarified by centrifugation at 3000 rpm for 15 min at 4 °C. The protein concentration of the supernatant was then determined by the Bradford assay. The activity of ChAT from each sample was determined spectrophotometrically using the assay kit from Nanjing Jiancheng Bioengineering Institute (Nanjing, China). Briefly, the assay medium containing CoA-SH as a substrate was incubated at 37 °C for 20 min. The activity was determined by measuring the absorbance at 412 nm.

### 4.8. Measurement of AChE Activity ex Vivo

For the analysis of AChE activity *ex vivo*, the brains of mice were dissected on ice into the frontal cortex and hippocampus. Samples were weighted, and a 10-times volume of lysis buffer was added (10 mM HEPES, pH 7.5, 1 mM EDTA, 1 mM EGTA, 150 mM NaCl and 0.5% Triton X-100). The homogenization was done by vortexing on ice for 15 min. The homogenates were clarified by centrifugation for 15 min at 3000 rpm at 4 °C. The assay medium contained 0.1 M Na_2_HPO_4_ (pH 7.5), 10 mM DTNB and 1 mM ATCI. Samples was prepared to give 0.5 μg/mL and incubated with 0.1 mM ethopropazine hydrochloride for 5 min to inhibit BuChE activity. The reaction was undertaken at 37 °C for 15 min. The activity was determined by measuring the absorbance at 412 nm.

### 4.9. Measurement of AChE Activity in Vitro

The colorimetric method, which was modified for use in 96-well-plates with a final volume of 200 μL, was used for the detection of AChE activity *in vitro* [37]. Briefly, brains of rats collected immediately after decapitation were used as the source of AChE. Brain was weighted, and a 10-times volume of lysis buffer was added (10 mM HEPES, pH 7.5, 1 mM EDTA, 1 mM EGTA, 150 mM NaCl and 0.5% Triton X-100). The homogenization was done by vortexing on ice for 15 min. The homogenates were clarified by centrifugation for 15 min at 3000 rpm at 4 °C. The assay medium contained 0.1 M Na_2_HPO_4_ (pH 7.5), 10 mM DTNB and 1 mM ATCI. The brain lysate was incubated with 0.1 mM ethopropazine hydrochloride for 5 min to inhibit BuChE activity. Test compounds were added to the assay solution and pre-incubated at 37 °C with the enzyme for 15 min followed by the addition of substrate. The activity was determined by measuring the absorbance at 412 nm. The inhibitory curve and IC_50_ were determined from a series of fucoxanthin concentrations.

### 4.10. Molecular Docking

Molecular docking analyses were accomplished by using the SYBYL (Tripos Inc., St. Louis, MO, USA) software and the programs embedded therein. The three-dimensional crystal structure of AChE complexed with donepezil was retrieved from the Protein Data Bank (PDB code: 1EVE) [38]. The three-dimensional structure of fucoxanthin was constructed using the standard geometric parameters of SYBYL and then optimized using the Powell method. The Surflex-Dock program, which uses an empirically-derived scoring function based on the binding affinities of protein-ligand complexes, was employed to perform docking analysis. As a flexible docking method, Surflex-Dock has been proven to be efficient in analyzing a variety of receptors [39]. The active site of AChE was defined relative to the coordinates of donepezil. During the simulations, the rotatable bonds of the ligands were defined, whereas the receptor was kept rigid. Standard parameters were used to estimate the binding affinity characterized by Surflex-Dock scores.

### 4.11. Data Analysis and Statistics

The data are expressed as the mean ± SEM. Statistical significance was determined by one-way ANOVA and Tukey’s or Dunnett’s test for *post hoc* multiple comparison, with the exception of mean escape latency, which was analyzed using two-way repeated-measures ANOVA followed by the LSD *post hoc* test. Differences were accepted as significant at *p* < 0.05.

## 5. Conclusions

In summary, we have found that fucoxanthin effectively reverses cognitive impairments in a scopolamine-induced mouse model. Moreover, fucoxanthin directly inhibits AChE by a non-competitive mechanism *in vitro*, possibly via interacting with the PAS of AChE. Based on these novel findings, we anticipate that fucoxanthin might provide significant therapeutic efficacy for the treatment of AD by acting on multiple targets, including inhibiting AChE and increasing BDNF expression in particular.

## Figures and Tables

**Table 1:** Fucoxanthin decreases AChE activity in the hippocampus and cortex of scopolamine-treated mice.

**Brain Region**	**AChE Activity**					
	Control	Scopolamine 3 mg/kg *i.p.*				
		Vehicle	Fucoxanthin (mg/kg)			Donepezil (mg/kg)
			50	100	200	3
Hippocampus	60.3 ± 2.7	80.2 ± 2.3 ^##^	76.2 ± 4.0	68.1 ± 3.2 **	67.6 ± 4.3 **	62.4 ± 2.6 **
Cortex	52.1 ± 2.8	71.2 ± 1.3 ^##^	60.5 ± 3.0^**^	62.4 ± 2.9 **	57.4 ± 3.2 **	61.6 ± 1.4 **

Data, expressed as the mean ± SEM (*n* = 8), are nanomoles of acetylcholine degraded/milligram protein/hour. ^##^ *p* < 0.01 *versus* the control group, ** *p* < 0.01 *versus* the scopolamine-treated group (ANOVA and Tukey’s test).

**Table 2:** Fucoxanthin increases ChAT activity in the hippocampus and cortex of scopolamine-treated mice.

**Brain Region**	**ChAT Activity**					
	Control	Scopolamine 3 mg/kg *i.p.*				
		Vehicle	Fucoxanthin (mg/kg)			Donepezil (mg/kg)
			50	100	200	3
Hippocampus	32.8 ± 1.1	27.6 ± 0.8 ^#^	30.0 ± 2.0	32.9 ± 1.6 *	30.5 ± 1.8 *	31.2 ± 1.8 *
Cortex	30.0 ± 1.3	25.2±1.0 ^#^	27.7 ± 1.9	34.0 ± 2.4 *	30.7 ± 1.5 *	33.7 ± 2.8 *

Data, expressed as the mean ± SEM (*n* = 8), are nanomoles of acetylcholine formed/milligram protein/hour. ^#^ *p* < 0.01 *versus* the control group, * *p* < 0.05 *versus* the scopolamine-treated group (ANOVA and Tukey’s test).
